# A Miniaturized Amperometric Hydrogen Sulfide Sensor Applicable for Bad Breath Monitoring

**DOI:** 10.3390/mi9120612

**Published:** 2018-11-22

**Authors:** Hithesh K. Gatty, Göran Stemme, Niclas Roxhed

**Affiliations:** Micro and Nanosystems, KTH Royal Institute of Technology, SE-100 44 Stockholm, Sweden; hithesh@kth.se (H.K.G.); stemme@kth.se (G.S.)

**Keywords:** hydrogen sulfide, amperometric, MEMS, gas sensor, bad breath, halitosis

## Abstract

Bad breath or halitosis affects a majority of the population from time to time, causing personal discomfort and social embarrassment. Here, we report on a miniaturized, microelectromechanical systems (MEMS)-based, amperometric hydrogen sulfide (H_2_S) sensor that potentially allows bad breath quantification through a small handheld device. The sensor is designed to detect H_2_S gas in the order of parts-per-billion (ppb) and has a measured sensitivity of 0.65 nA/ppb with a response time of 21 s. The sensor was found to be selective to NO and NH_3_ gases, which are normally present in the oral breath of adults. The ppb-level detection capability of the integrated sensor, combined with its relatively fast response and high sensitivity to H_2_S, makes the sensor potentially applicable for oral breath monitoring.

## 1. Introduction

Bad breath or oral malodor, affects a majority of the population on a regular basis. The presence of plaque, tongue coating [1], gum diseases [2], exposed necrotic tooth pulp, and healing wounds [3] are known to be the cause of oral malodor. Microorganisms present in oral cavities react with organic compounds, releasing sulfur-containing by-products that lead to bad breath. Specifically, sulfur-containing by-products, such as hydrogen sulfide (H_2_S), methyl mercaptan (CH_4_S), and dimethyl sulfide ((CH_3_)_2_S), are associated with bad breath, which is also termed as halitosis [4].

Until recently, oral malodor was diagnosed by physicians in a purely subjective manner (smelling). However, recent developments in sensor technology have provided measuring instruments with sensitive detection of bad breath. The most successful commercial measuring instrument is the Halimeter™ [5], a standard clinical bench-top instrument used to measure volatile sulfur compounds (VSCs), particularly H_2_S gas concentration. In this instrument, the user blows into a tube attached to the instrument and a concentration value is presented on a display. Halitosis in an adult is classified as “normal” if the concentration is within the range of 80–160 parts-per-billion (ppb), “weak” if the concentration is within the range of 160–250 ppb, and “strong” if the concentration is greater than 250 ppb [6,7]. The disadvantage of the Halimeter instrument is that it is a bench-top apparatus (3.6 kg) that requires warm-up times and yearly maintenance [8] and is thus an instrument that is primarily designed for patient examination or population studies. However, to more directly address and counteract personal discomfort, ad-hoc mobile monitoring of bad breath would be highly desired. To achieve such monitoring, the sensor element is essential, which requires a small form factor for integration, a fast response time, and ppb-level sensitivity.

Among the various types of H_2_S sensors developed, amperometric sensors are particularly advantageous as it allows the fabrication of miniaturized and high sensitivity sensors with fast response time. Schiavon and Zotti achieved detection limits of 45 ppb using discrete porous silver electrodes supported on separate ion-exchange membranes [9]. However, nonintegrated discrete components result in relatively large-size sensors, which is undesirable when developing a handheld instrument. Recently, Yang et al. showed a fast response Nafion-based amperometric sensor that could detect H_2_S in the range of 0.1–200 ppm [10]. However, a complex fabrication method of the sensing electrode and lower sensitivity limits the sensor from being used in the ppb range, which is required for bad breath detection.

In the present work, a miniaturized and integrated electrochemical H_2_S sensor with fast response time and a ppb-level sensitivity that is applicable for Halitosis measurement is demonstrated. A simple fabrication method involving high aspect ratio etching and atomic layer deposition of platinum provides the basic structure for preparation of the sensing electrode. The sensor was characterized for its cross sensitivity to nitric oxide (NO), which is normally present in the oral cavity and nasal cavity. Nasal cavity NO contributes to the high concentration in the oral cavity and can affect the NO concentration in the oral cavity. A typical concentration of NO in the nasal cavity is in the range of 0–900 ppb [11], while it is in the range of 20–100 ppb in the oral cavity. In addition, the sensor was characterized with ammonia (NH_3_) gas, which is present in the oral cavity in the range of 0–450 ppb [12].

## 2. Sensor Design and Measurement Method

**Figure 1:**
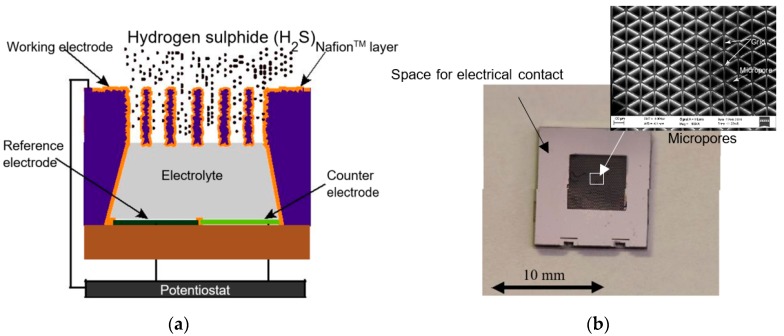
(**a**) Schematic cross section of the sensor design indicating the high aspect ratio micropores with a nanostructured porous Nafion™ layer. (**b**) Photograph of the amperometric sensor with the porous structure in the middle (dark area). Inset: SEM image shows the microporous grid structure of the working electrode. The working electrode of the sensor is fabricated by deep reactive ion etching and platinum atomic layer deposition of a silicon on insulator (SOI) wafer, and the counter and reference electrodes are fabricated on a glass wafer, which is then assembled together by anodic bonding [13]. The sensor has a footprint area of 10 × 10 mm^2^ and a thickness of approximately 1 mm.

The sensor design is based on the principle of amperometric detection of H_2_S gas. The working, reference, and counter electrodes, together with the electrolyte, constitute the basic elements of the sensor. Particularly in the present design, the working electrode consists of a nanostructured Nafion™ (Chemours, Wilmington, DE, USA) coating that in turn is leveraged through a microporous high aspect ratio structure. The interaction between this large-area working electrode, the gas, and the electrolyte (5% H_2_SO_4_) under electrical bias leads to the oxidation of H_2_S gas at the surface of the electrode, causing a current flow between the working and the counter electrodes. The working electrode current is then measured using a potentiostat, maintaining a constant voltage of +1.1 V with respect to the reference electrode. Figure 1a shows the schematic cross section of the sensor design, and Figure 1b shows the photograph of a bare die of the sensor with a dimension of 10 mm × 10 mm × 1 mm. The design, fabrication, and assembly of the sensor have been described in our previous work [13].

**Figure 2:**
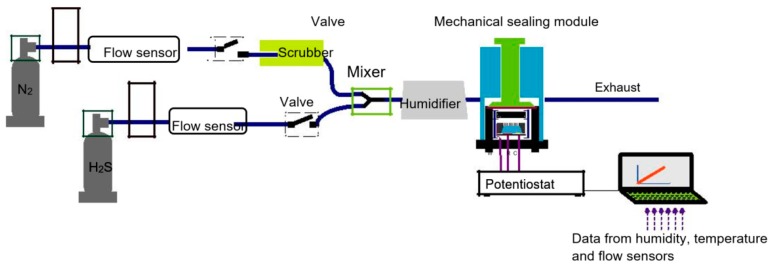
Schematic illustration of the measurement set-up used for characterization of the H_2_S amperometric sensor. Data from the flow sensors, the temperature sensor, and the humidity sensor were accessed using a LabVIEW ™ (National Instruments, Austin, TX, USA) program.

In order to test the sensor for different gases and gas concentrations, a measurement set-up was built as illustrated in Figure 2. In this set-up, a 10 ppm H_2_S in N_2_ gas (AGA gas AB, Lidingö, Sweden) was mixed with a pure N_2_ gas (99.95% pure, AGA gas AB, Lidingö, Sweden) to obtain the desired concentration. A scrubber (Dräger, type 1140, Lidingö, Sweden) was used to remove potential residues in the N_2_ gas. To measure the selectivity of the sensor to interfering gases, 200 ppb NO in N_2_ gas (AGA gas AB, Lidingö, Sweden) and 45 ppm of NH_3_ in N_2_ gas (AGA gas AB, Lidingö, Sweden) were used. In order to humidify the gas mixture, a custom-made humidifier consisting of a syringe with moistened paper was used. A mechanical sealing module was further used to reduce evaporation of the electrolyte. Further details on the measurement set-up used for sensor characterization can be found in our previous work [13].

## 3. Results and Discussion

The sensor was tested for its H_2_S gas sensitivity, selectivity to NO and NH_3_, and response time. A gas flow of 550 mL/min and 50% relative humidity (RH) was maintained for all measurements.

### 3.1. Sensitivity

**Figure 3:**
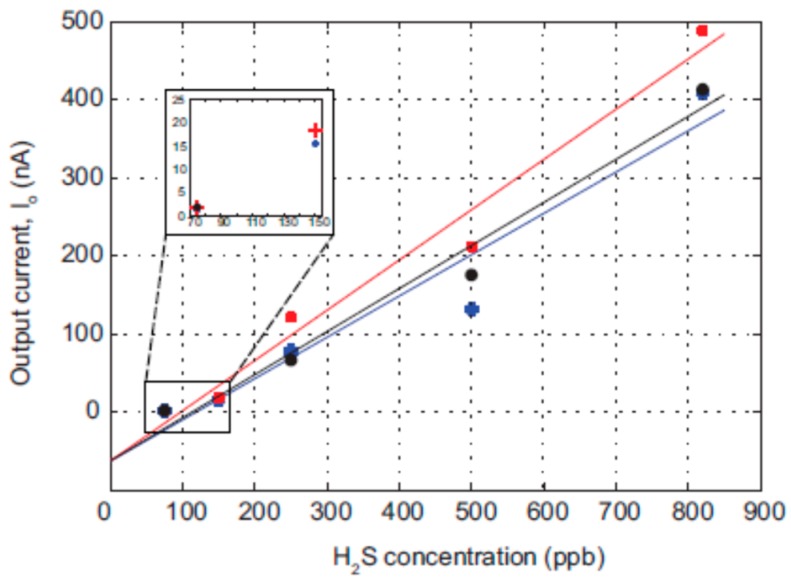
Output current I_o_ as a function of H_2_S concentration. A linear fit to each concentration variation gives the slope, which is then related to the sensitivity of the sensor.

In order to determine the sensitivity of the sensor, the H_2_S concentration was varied in five steps of 75, 150, 250, 500, and 820 ppb. Three such variations were carried out using the measurement set-up. The output current from each concentration was determined by calculating the difference between the working electrode current at t_90_ (cf. Figure 5) and the working electrode current at zero H_2_S concentration. The output currents and the linear fitting for five different H_2_S gas concentrations are plotted in Figure 3. Based on the linear fit, the maximum sensitivity of the sensor was calculated to be 0.65 nA/ppb. The sensor detects H_2_S gas in the lower limit of 75 ppb and a higher limit of 820 ppb and is within the range required for monitoring the oral breath. The lower limit concentration of 75 ppb was measured without being affected by the noise in the system.

**Figure 4:**
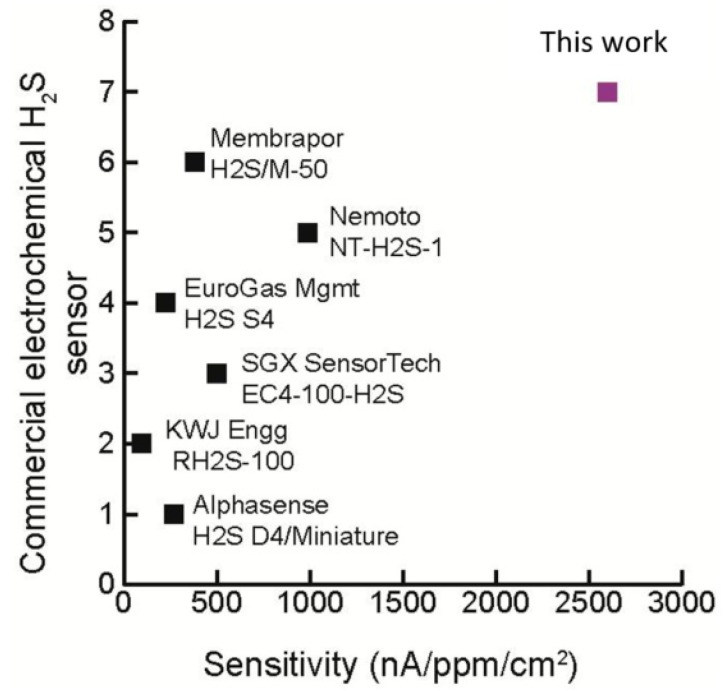
Comparison of area-normalized H_2_S sensitivity ranges for several commercial amperometric sensors. The sensitivity of the integrated sensor is approximately 2.5 times higher than the sensitivity of commercial sensors.

The sensitivity graph of commercial H_2_S sensors as compared to the designed integrated sensor is shown in Figure 4. In order to maintain a fair comparison, the area of the working electrode is normalized to the sensitivity, i.e., 0.65 nA/ppb is obtained for a footprint area of 25 mm^2^ arriving at a normalized sensitivity of 2700 nA/ppm/cm^2^. The integrated H_2_S sensor has a measured area normalized sensitivity that is approximately 2.5 times more than the sensitivities of commercial sensors. This shows a potential for the integrated sensor to be fabricated with a smaller footprint area with a reduced sensitivity in order to realize a smaller sensor, leading to a miniaturized handheld instrument.

### 3.2. Selectivity to NO and NH_3_

To obtain the selectivity to NO gas, the output current was measured at 200 ppb NO gas concentration, and the NO sensitivity was calculated to be approximately 0.04 nA/ppb, which is in agreement with our earlier fabricated prototype [13,14]. The selectivity can be defined as the ratio of H_2_S sensitivity to NO sensitivity and is calculated to be approximately 16. NO concentration of approximately 900 ppb is commonly found in the nasal cavity that can affect the oral breath [11]. However, a concentration of 900 ppb of NO results in an equivalent H_2_S concentration of 55 ppb, which is within a normal halitosis range. Therefore, the NO contamination from the nasal cavity has negligible effect on the H_2_S concentration from the oral breath. The selectivity of the sensor to NO could be further increased by reducing the nasal NO contamination from the oral cavity either by breath maneuver or by clamping the nostrils while measuring H_2_S concentration from the oral cavity. Other sources of NO release, such as lungs and oral cavity, can be neglected due to a low NO concentration (20–100 ppb), which is likely to have a minimal interference with H_2_S detection.

The output current of the sensor to 45 ppm NH_3_ gas concentration was found to be below the detection limit. Therefore, the sensor will not be sensitive to NH_3_ gas present in the oral cavity.

### 3.3. Response Time

**Figure 5:**
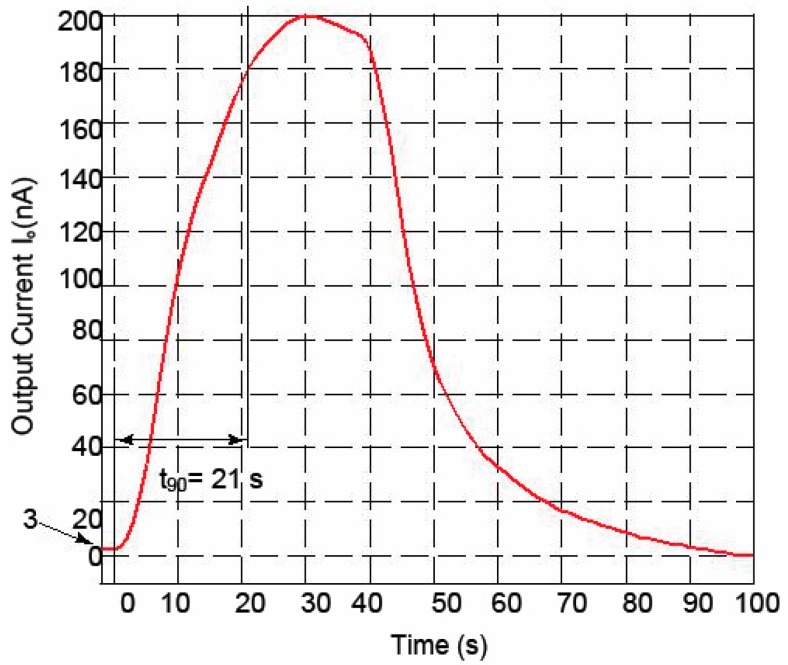
Output current response to 250 ppb step of H_2_S gas concentration. A response time (t_90_), i.e., the time required to reach 90% of the maximum output current, of 21 s was measured for the sensor.

The response time of the sensor to a 250 ppb H_2_S concentration was estimated by measuring the rise time (t_90_) of the sensor, i.e., the time required to reach 90% of the maximum output current. The response time of the sensor was measured to be 21 s, as shown in Figure 5. A thorough investigation of the method of oral breath sampling to capture the H_2_S gas concentration at a constant rate that could be correlated to the sensor response time has not yet been reported. However, Tangerman et al. collected a sample of oral breath by breathing into a syringe for 5–10 s. The sample was then used for H_2_S concentration detection [15]. The recommended procedure for Halimeter instrument includes an initial three-minute period during which the patient breathes through the nose with lips sealed. A pipe attached to the instrument is then inserted into the partially opened mouth, and a pump withdraws gas from the oral cavity for concentration measurement [16,17]. It is conceivable that an oral breathing in the range of 20–30 s could be considered for H_2_S concentration measurement.

**Figure 6:**
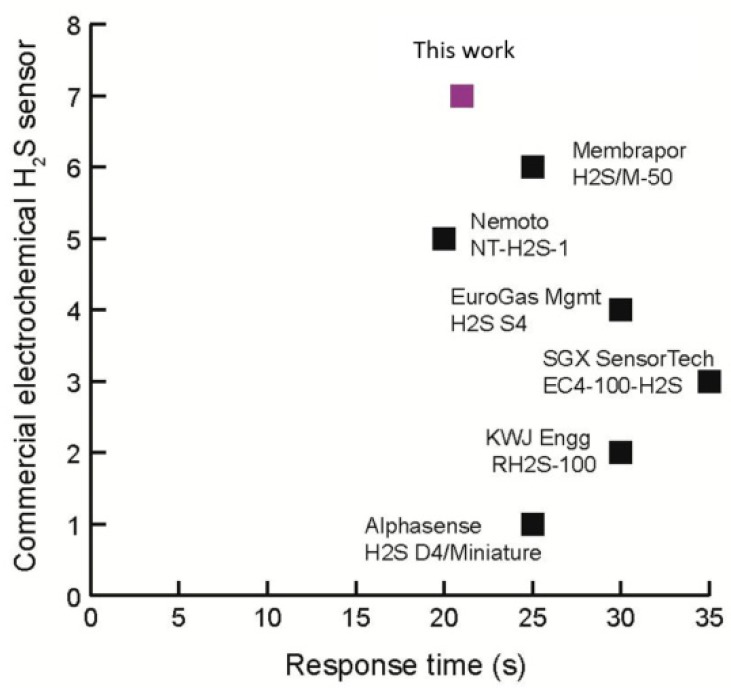
Response times of several commercial amperometric H_2_S sensors. The graph shows that the integrated sensor has a better response time compared to commercial sensors.

In order to assess the response time of the integrated sensor, a comparison graph with commercial H_2_S sensors is shown in Figure 6. The response time (t_90_) of 21 s is comparatively better than most of the currently available commercial sensors. In order to have a real-time measurement, the response time can be further decreased by optimization of the sensor design.

### 3.4. Working Electrode Current Drift

**Figure 7:**
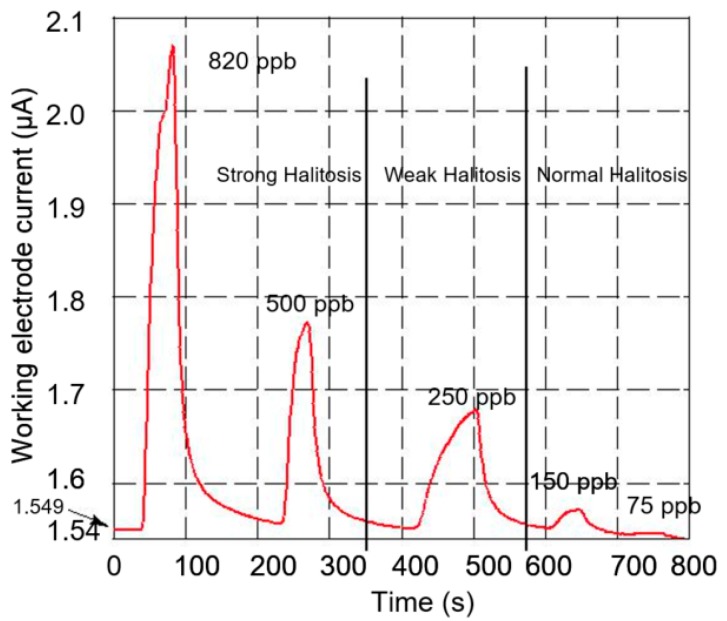
Working electrode current for five different H_2_S concentrations. The sensor is able to detect the entire dynamic range of H_2_S concentration that could be correlated to the concentration present in the oral breath. The background current of the sensor could be due to the interference from humidity or due to high conductivity of the electrolyte.

The response of the sensor to five different concentrations is shown in Figure 7. The graph shows a background current drift of 0.675 nA/min, which is equivalent to approximately 1 ppb/min of H_2_S concentration drift. Therefore, for a response time of 21 s, the drift component is approximately 0.3 ppb, which is negligibly small. Hence, an accurate measurement of H_2_S concentration can be deducted from the output current.

### 3.5. Halitosis Measurement Range

Figure 7 shows the working electrode current for five different steps of H_2_S gas concentration. The sensor can measure the H_2_S concentration in the region of normal, weak, and strong halitosis that is required for monitoring the oral health. Consequently, the sensor can be applied to detect the entire dynamic range of H_2_S concentration present in the oral breath. In addition, the sensor could be useful in dental clinics for follow-up measurement of the halitosis content before and after oral dental treatment.

## 4. Conclusions

In this paper, an integrated amperometric sensor has been evaluated for the detection of hydrogen sulfide (H_2_S) concentration present in the oral breath. The sensitivity of the sensor is measured to be 0.65nA/ppb with a response time of approximately 21 s, which is comparatively better than commercially available sensors. The sensor can be applied to measure bad breath where the concentration of H_2_S gas indicates a malodor in the breath. The entire range of H_2_S gas concentration present in the oral breath can be diagnosed by the sensor. For more complete measurements, detection of methyl mercaptan (CH_4_S) and dimethyl sulfide (CH_3_)_2_S concentration in combination with H_2_S concentration will give a more comprehensive representation of oral health. Overall, a prototype has been realized to measure H_2_S gas concentration that is relevant for bad breath monitoring.
